# Listening to Limericks: A Pupillometry Investigation of Perceivers’ Expectancy

**DOI:** 10.1371/journal.pone.0074986

**Published:** 2013-09-23

**Authors:** Christoph Scheepers, Sibylle Mohr, Martin H. Fischer, Andrew M. Roberts

**Affiliations:** 1 Institute of Neuroscience and Psychology, University of Glasgow, Glasgow, United Kingdom; 2 Department of Psychology, University of Potsdam, Potsdam, Germany; 3 School of Humanities, University of Dundee, Dundee, United Kingdom; University of Leicester, United Kingdom

## Abstract

What features of a poem make it captivating, and which cognitive mechanisms are sensitive to these features? We addressed these questions experimentally by measuring pupillary responses of 40 participants who listened to a series of Limericks. The Limericks ended with either a semantic, syntactic, rhyme or metric violation. Compared to a control condition without violations, only the rhyme violation condition induced a reliable pupillary response. An anomaly-rating study on the same stimuli showed that all violations were reliably detectable relative to the control condition, but the anomaly induced by rhyme violations was perceived as most severe. Together, our data suggest that rhyme violations in Limericks may induce an emotional response beyond mere anomaly detection.

## Introduction

Poetry is a literary art form that is enjoyed throughout the world. Poems are characterized by systematic design features that tend to evoke a complex set of cognitive, emotional, and evaluative processes. These design features can include visual layout, metre, rhyme scheme, alliterations, figurative language, and many others. Poetic metre is a dominant design feature, determining line length and imposing restrictions on grammar and lexical choices in order to create a specific poetic rhythm [1] (p. 134). Originally, poems were meant for oral presentation to a listening audience, and several of their features, such as rhyme and imagery, are known to support both retrieval from and encoding into memory (see below).

The normal critical understanding of poetry is that with regard to form, it works through a combination of fulfilled expectations and elements of surprise. So, for example, a classic English poetic form such as Shakespeare’s dramatic blank verse (unrhymed iambic pentameters) has an underlying pattern defined by the sequence and number (per line) of stressed and unstressed syllables. Audience members or readers of the plays hear these regularities and come to expect a continuation of the pattern; but complete regularity would be monotonous and undramatic (a condition approached by some minor Elizabethan drama). Shakespeare used variations, such as (commonly) substituting a spondee (two stressed syllables) or trochee (stressed then unstressed) for one or more of the iambs (unstressed then stressed). Another common variation is a ‘feminine ending’ (an extra syllable at the end of a line). There are other variations, of which some are relatively subtle. Also, it should be noted that the ‘stressed’ vs. ‘unstressed’ distinction is a conventional simplification for the purpose of pattern-identification: in practice there are various degrees of stress. Variations in the pattern serve dramatic, rhetorical and poetic purposes, emphasizing particular words, conveying mood or tone, or generating aural or semantic effects.

The expectation of pattern is a source of poetic pleasure (and therefore emotion) in itself, but so is variation. Similar principles apply, in rhymed poetry, to the substitution of half-rhymes for full rhymes. The general principle of pattern with variation can be applied to most forms of poetry, including that in other languages, even if the patterns are formed of different elements. Limericks would have to be regarded as a special case, being comic verse rather than poetry, with exceptionally strong expectations for rhythm, rhyme and semantic pattern. Its meaning often takes the form of a joke, but sometimes that of bathos.

Experimental evidence can inform this literary debate about poetry reception. For example, Hanauer [2] investigated which features of a text suggest to readers that it is a poem. He asked readers to rate the degree of ‘poeticity’ of graphically manipulated poems (capitalization, spacing) and of phonetically manipulated poems (alliteration, consonance, assonance and rhyme). Participants assigned higher ratings of ‘poeticity’ to materials where these features were most salient. Hanauer concluded that readers make a genre decision about each text they encounter and that this influences the way any remaining information is processed.

Carminati and colleagues [3] studied whether expectancy of a particular rhyme scheme develops during silent reading. They presented their participants with several randomly ordered stanzas from one of two poetic sub-genres: ottava rima poems by Byron [4] or elegies by Gray [5]. Both types of poems make use of regular rhymes at the end of lines and are written in iambic pentameter, but the rhyme scheme for ottava rima is ABABABCC whereas the rhyme scheme for the elegies is ABAB CDCD. Importantly, after seven stanzas of one rhyme scheme, each reader received three further stanzas from the other rhyme scheme. Self-paced reading times were recorded for each line of text but did not show a systematic slowing after a switch in the rhyme scheme. Carminati et al. tentatively concluded from this null result that readers do not necessarily focus on rhyme schemes while reading poems.

Another study documented how alliterations enhance text recall [6]. Three groups of participants read texts containing either a recall-cue *consistent* type of alliteration (e.g., “all along the way-winding road, wary whispers of the old barn,”), a recall-cue *inconsistent* type of alliteration (e.g., “all along the raw and rutted road the reddish barn,”), or a line containing the same concepts but in a non-alliterative format (e.g., “all along the creek-winding road, past Stuart's barn,”). After reading ten more lines of text, all participants encountered a cue line (“the wooden willowy warp of wild carrot”), and then saw a probe word (e.g., “barn”) which they had to rapidly classify as either “old” (i.e., the word was mentioned in the previous text) or “new”. Note that, in this example, the correct answer for all participants was “old”. Interestingly, participants in the first group responded faster than the other two groups, presumably because the w-based alliteration in the cue line was consistent with the w-based alliteration during encoding and thus re-instantiated the associated concept of “barn”. The result held regardless of whether reading was silent or aloud, and also regardless of text format (poetry or prose). This observation helps to explain how specific poetic features support cognitive mechanisms, even outside of their typical genre. It also shows how the study of poetry can, in turn, reveal elementary aspects of human cognition which poets have learned to use (cf. [7]).

The present study follows on from such empirical investigations of poetry reception, focussing specifically on the cognitive processing of Limericks. Limericks are five-line poems that are characterized by a strict AABBA rhyme scheme and often humorous content [8]. A number of basic issues about the cognitive processing of poems can be addressed with Limericks. Specifically, we evaluated four types of recipient expectations, namely semantic, syntactic, rhyme, and metric expectancies. Let us consider each type of expectation in turn.

Violations of semantic expectancy have been extensively investigated in the psycholinguistic literature. In a landmark study, Kutas and Hillyard [9] recorded electrical brain activity (event-related potentials or ERPs) during passive listening to sentences which sometimes contained semantic violations of the form “He spread the warm bread with socks”. This allowed the authors to determine the time-course of semantic anomaly detection. They discovered a negative deflection of electrical activity that signalled rapid detection of the anomalous sentence completion within about 400 ms. Hoorn [10] subsequently reported an ERP study on how readers process poems that ended with either semantic or rhyme violations or both. He found interactive effects of both types of violations on the activity pattern, such that the semantic effect was more clearly expressed when rhyme was also violated.

Syntactic violations (e.g., non-canonical or even ungrammatical word orders) have also been studied extensively by psycholinguists, but very rarely so in the context of poetry reception. Thus, we added a syntactic violation condition to our experiments to explore the relative importance of syntax as compared to other features of a poem. Perceivers may be more tolerant to syntactic violations in poetry than in everyday prose, as poets often deliberately scramble syntax beyond what is normally considered grammatical (e.g., “Agree I could not” instead of “I could not agree”) to achieve a certain effect in the perceiver.

The processing of certain types of poetry is also characterized by the perceiver’s expectation of a rhyme scheme. Carminati et al. [3] found relatively little evidence for rhyme scheme expectations to matter during processing when contrasting two relatively similar rhyme schemes (ottava rima and elegy) during silent reading. However, Limerick recipients should strongly expect the last line of the Limerick to rhyme with the first two, thus allowing for a simpler and potentially stronger test of rhyme expectancy compared to [3]. Another difference to [3] is that we presented our stimuli in the auditory modality to further enhance rhyme-related phonological processing.

Finally, the present study explored the cognitive processing of rhythmical structure, specifically the processing of metre violations. Rhythmic structure is another highly regular feature of Limerick poems which are characterized by stress being placed on the last or second-to-last syllable of the last word of the Limerick. By violating this specific construction rule of the Limerick, we interfered with metre expectancy which might become evident in increased processing effort (cf. [11]).

As indicated above, a continuous measure of on-line processing is particularly useful in the assessment of expectancy violations. Instead of the ERP method, we adopted the continuous recording of pupil size, another spontaneous biological signature of nervous system activity. To our knowledge, the present study is the first that tests whether pupillary responses are sensitive to anomaly detection in poetry.

Pupillary responses are regulated by the autonomic nervous system which is not under direct voluntary control. However, the measure has been found to be a sensitive indicator of cognitive load, showing increases in pupil diameter for tasks with increased mental effort (e.g., [12]) which are fast enough to be detectable during on-line language processing [13], [14]. Pupillary responses are also known to reflect increased affective arousal independent of emotional valence [15], making them potentially very useful in the context of poetry processing. Importantly, since the pupil also responds to changes in ambient illumination [16], we took great care to prevent such confounds by presenting all our materials in the auditory modality while holding illumination constant.

## Experiment 1

The purpose of Experiment 1 was to establish pupil diameter as a means of assessing expectancy violations during spoken Limerick appreciation.

### Method

#### Participants

Forty native speakers of British English were recruited from the Dundee University undergraduate pool. All participants were right-handed, with normal hearing and normal or corrected-to-normal vision. Participants were paid £3 each. A typical session lasted about 25 minutes. All participants reported to be familiar with Limericks and none reported to have had above-average exposure to (or expertise of) poetry.

#### Ethics statement

The research was approved by the University of Dundee Research Ethics Committee and participants gave written informed consent prior to taking part.

#### Design and materials

There were 25 spoken Limerick items (for transcripts and audio recordings, see Appendix S1), each appearing in five different conditions, as shown in the following example (the indices “_1_ **|**”, “_2_ **|**” and “_3_ **|**” mark critical time-points which will be explained in the Analysis section below):

*As Londoners say, in Calcutta*

*lives a man with a terrible stutter.*

*When he asks for the bread,*

*They will pass him instead:*

(1) *Beer, broccoli, beans*_1_ ***|*** *and the*_2_ ***|*** *butter*_3_ ***|.***

(2) *Beer, broccoli, beans*_1_ ***|*** *and the*_2_ ***|*** *gutter*_3_ ***|.***

(3) *Beer, broccoli, beans*_1_ ***|*** *some and*_2_ ***|*** *butter*_3_ ***|.***

(4) *Beer, broccoli, beans*_1_ ***|*** *and the*_2_ ***|*** *biscuits*_3_ ***|.***

(5) *Beer, broccoli, beans*_1_ ***|*** *and also*_2_ ***|*** *butter*_3_ ***|.***

As shown in the example, experimental conditions were manipulated towards the end of the last line per Limerick. The conditions were: (1) a control condition (no violations, i.e. the Limerick was spoken in its originally intended form), (2) a semantic violation condition (i.e. the last word did semantically not fit into the prior context), (3) a syntactic violation condition (typically involving a word-order violation before the last word), (4) a rhyme violation condition (i.e. the last word violated the AABBA rhyme scheme of the Limerick), and (5) a metric violation condition (i.e. a violation in rhythm, by adding [as in the example] or subtracting a syllable to/from the original verse). Semantic and rhyme violations mostly occurred in the position of the last word, whereas syntactic and metrical violations were typically introduced one or two words before the last word. Care was taken to ensure that the violation manipulations were orthogonal, i.e. without being confounded with other types of violations.

The stimuli were spoken by the last author (AMR), a male native speaker of British English. All stimulus versions were recorded in one session. We used cross-splicing to ensure that the introductory context before the critical final sentence was identical across the five versions per item. Using a Latin square rotation scheme, five different presentation lists were prepared such that (a) each Limerick item occurred exactly once per presentation list, (b) item-condition combinations were counterbalanced across presentation lists, and (c) each presentation list contained five Limerick items per condition. Each list was seen by eight participants. Also included in each presentation list were 13 ‘filler’ Limericks (recorded from the same speaker) which did not contain any violations, similar to the control condition. Thus, each participant was exposed to 18 Limericks without violations (5 control condition items and 13 fillers), plus five Limericks in each of the four (semantic, syntactic, rhyme, and metric) violation conditions. The stimuli per presentation list appeared in a quasi-random order, with two filler stimuli as warming-up trials at the beginning of each list.

#### Apparatus

Participants’ eye-positions, blinks, and pupil sizes (measured in *log numbers of pixels per video frame*) were continuously monitored using an SR-Research EyeLink II head-mounted eye-tracker (0.01° spatial resolution) running at 500 Hz sampling rate (one sample every 2 ms). Viewing was binocular, but only the participant’s dominant eye was tracked (the right eye for 29 participants, as established via a simple parallax test prior to the experiment). The display screen was a 21-inch CRT monitor running at 120 Hz refresh rate with 1024×768 pixels resolution. The Limerick audio files were played from a low-latency ASIO sound card connected to the display-PC of the eye-tracker. The stimuli were delivered to participants via a pair of ear-clip headphones.

#### Procedure

At the beginning of each experimental session, the participant was seated in front of the screen while the experimenter set up the headphones and eye-tracker cameras. The participant then performed a simple eye-tracker calibration task in which they had to look at nine different fixation targets on screen, plus another nine fixation targets for validation purposes. Initial setup and calibration usually took about one minute. Calibration was repeated at least once halfway through the experiment, or if the experimenter noticed a decline in accuracy (e.g. after a change in the participants posture). Each trial started with the presentation of a fixation target in the center of the screen (a black cross presented on a light-grey background). Participants fixated the target while the experimenter performed a semi-automatic drift correction. Three-hundred milliseconds after drift correction, the Limerick audio file started playing. The participant listened to the Limerick while continuing to look at the fixation target, which stayed on screen until at least 1500 ms after the Limerick ended. Then, after a blank screen period of 500–1500 ms (varying at random), either the next trial was initiated or (in about 25% of the trials), the Limerick was followed by a simple yes/no comprehension question (e.g. *Did the man stutter?*), printed in 24-point font on the screen. Participants had to answer these questions using either the left or the right arrow-key of a standard PC keyboard in front of them. The questions were included to ensure that participants paid attention to the Limericks’ contents; all participants answered at least 70% of the questions correctly. Answering the question triggered the presentation of the next trial. Participants were instructed to avoid head movements and eye-blinks while listening to the Limericks. They were encouraged to perform eye-blinks at the beginning of each trial (i.e. before drift correction).

#### Analysis

For each experimental item, and in each condition, we defined the *critical* word onset as the onset of the earliest word position from which on conditions started to differ (critical word onsets are indicated with “_1_ **|**” in the *Design and Materials* example). Since it was impossible to manipulate all types of violations in exactly the same word position, we opted for this kind of synchronization and compared pupil size changes over a relatively long period of time thereafter. We also measured, relative to the critical word-onset, the onset of the final word (“_2_ **|**”) and the offset of the Limerick (“_3_ **|**”). For each trial, we then defined a 2200 ms analysis window, starting from 200 ms (100 eye-tracker samples) before the critical word onset and ending 2000 ms (1000 eye-tracker samples) after the critical word onset. Only pupil size data within that time window were considered. Although participants were instructed to avoid eye-blinks during trials, a pre-screening of the data indicated that they were unable to do so in 37.2% of the trials, suggesting that such events often happen involuntarily (indeed, blinks were unsystematically distributed over time and conditions, and there were large inter-individual differences in their frequency of occurrence). Removing these occasional blinks (plus 8 eye-tracker samples before and after each blink) from the affected trials resulted in time periods with missing pupil size data, which ranged ca. 50–200 ms in length. To fill the resulting “gaps” in the continuous pupil-size output per trial, we employed a non-linear data interpolation method (2^nd^ order B-spline estimation after Loess smoothing over 3% data windows; for an illustration, see Appendix S2). If a trial did not comprise missing pupil size data within the critical 2200 ms time period (62.8% of the cases), no smoothing/interpolation was applied. Next, we subtracted the mean pupil size across the first 100 samples within the critical time window from the remaining 1000 samples (normalisation). The data reported below therefore reflect deviations from the *baseline* pupil size established during the 200 ms time period before the critical word-onset.

### Results

**Figure 1:**
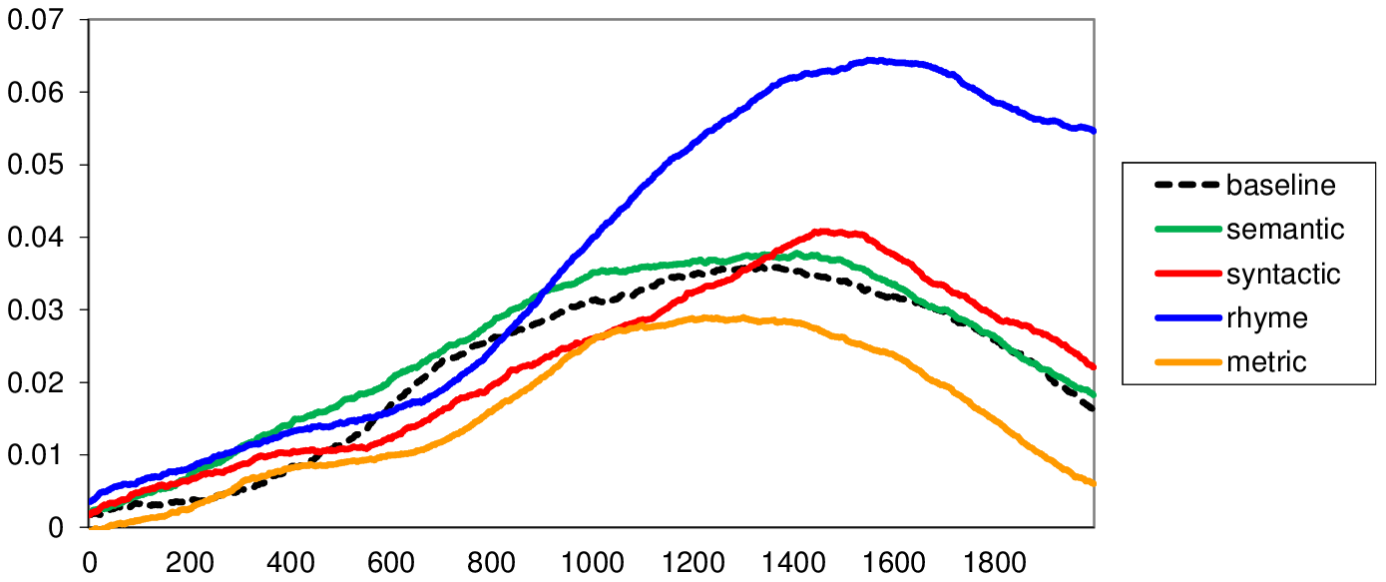
Pupil size per condition, from 0–2000 ms after critical word-onset. Pupil size (Y-axis) is measured in log number of pixels per video frame, relative to a 200 ms pre-onset baseline. Time (X-axis) is sampled at 500 Hz.

**Figure 2:**
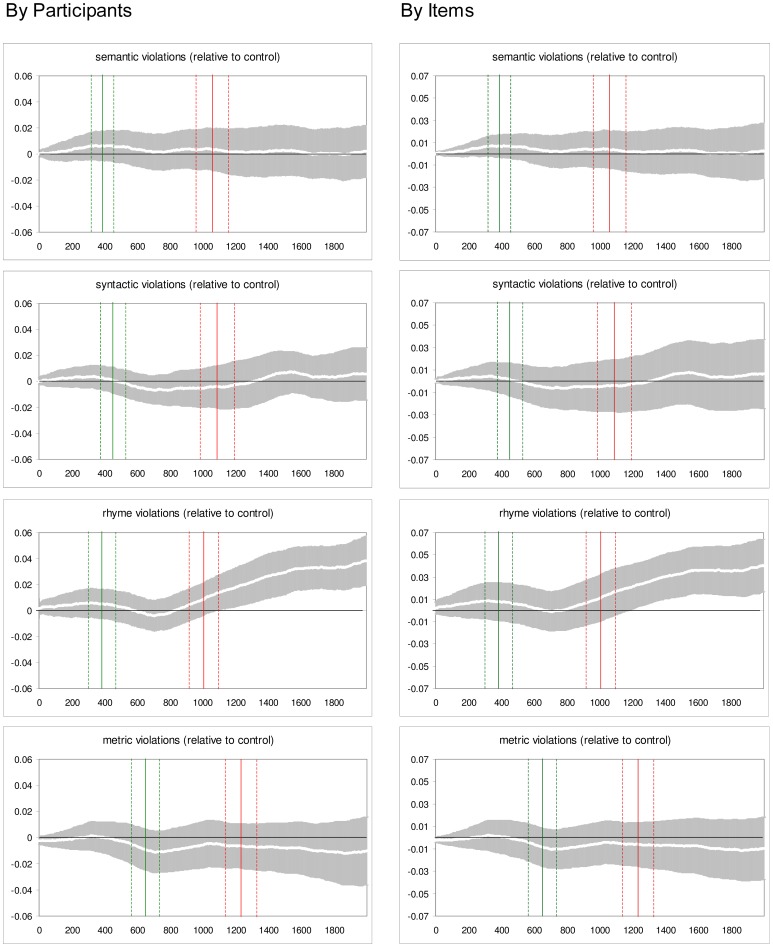
Pupil size deviations from the control condition (no violation) as a function of time. Time is plotted on the X-axis, from 0–2000 ms after critical word onset. The white curve in each plot refers to the mean difference between the relevant violation condition and the control condition (higher values mean more dilated pupils in the violation condition); the grey areas around the curves indicate 95% confidence bands for the difference. The green vertical line in each plot marks the average onset of the final word in the given violation condition, together with 95% confidence limits (green dotted lines). Red solid and dotted lines mark the average Limerick offset (end of auditory presentation) and corresponding 95% confidence limits. Left hand panels: by participants; right hand panels: by items. Top to bottom: semantic, syntactic, rhyme, and metric violation.

As can be seen in Figure 1, the rhyme violation condition clearly stood out from the remaining conditions by being associated with more dilated pupils. The effect appeared from ca. 1000 ms after critical word-onset until the end of the considered time period. We averaged the pupil size data over this time interval (1000–2000 ms after critical word-onset) and entered them into one-way ANOVAs with *condition* as a repeated-measures factor (*F*_1_ for data aggregated up to the participant level, *F*_2_ for data aggregated up to the item level). The analyses showed a clear effect of condition: *F*_1_(4, 156) = 3.85; *Greenhouse-Geisser Epsilon* = .84; *adjusted p*<.01; *F*_2_(4, 96) = 3.10; *Greenhouse-Geisser Epsilon* = .80; *adjusted p*<.03. Comparisons with the control condition (paired *Bonferroni* t-tests) confirmed that the rhyme violation condition elicited a significant pupillary response (*p*s <.05), whereas the other types of violations did not (*p*s >.5). Figure 2 shows continuous plots of the pupillary response in each violation condition relative to the control condition (without violation); it confirms the previously established pattern and indicates that the pupillary response in the rhyme violation condition reached significance (by participants and items) from about 200 ms after the offset of the final word.

### Discussion

Pupillary responses were found to be sensitive to expectancy violations during Limerick appreciation. Contrary to the null results reported in [3], pupillary responses were selectively sensitive to rhyme violations, indicating that poetry recipients pay attention to this feature. However, pupillary responses were not sensitive to semantic, syntactic, or metrical violations. There are at least two possible explanations of this result. On the one hand, rhyme violations in Limericks might evoke a qualitatively unique response when compared to the other types of violations. Alternatively, rhyme violations might simply be the only clearly noticeable type of violation in the context of a Limerick. An anomaly-rating study was conducted to assess these possibilities.

## Experiment 2

The main goal behind the second experiment was to determine whether each type of violation (syntactic, semantic, rhyme, and metric) was reliably detectable in comparison to the control (no violation) condition.

### Method

#### Participants

Twenty new participants (native English speakers) were recruited from the University of Glasgow undergraduate pool in exchange for course credits. All participants reported to be familiar with Limericks and none reported to have had above-average exposure to (or expertise of) poetry. A typical session lasted about 15 minutes.

#### Ethics statement

The research was approved by the University of Glasgow College of Science and Engineering ethics committee. Participants gave written informed consent prior to taking part.

#### Design and materials

Experimental and filler items consisted of the same set of spoken Limericks as in the previous experiment. Order of items was randomized individually per participant, with two fillers as warming-up trials at the beginning. There were five different presentation lists (each seen by four participants) containing 25 experimental items (five per condition per list, with different item-condition combinations across lists) and 13 fillers.

#### Apparatus

A standard PC with keyboard and headphones was used. The experiment was controlled using DMDX software [17].

#### Procedure

The participants’ task was to rate the acceptability of each Limerick on a bipolar scale ranging from 1 (“perfectly ok”) to 7 (“highly anomalous”). In each trial, participants had to look at a fixation cross in the center of the screen while the spoken Limerick was played via headphones. After the audio file finished playing, the fixation cross was replaced with the 7-point scale, and participants had to provide their judgment by typing a number between 1 and 7 into a computer keyboard in front of them. They were informed that a higher number was meant to reflect “greater strength or certainty” of a perceived anomaly. Typing in a number triggered the presentation of the next trial.

#### Analysis

The participants’ ratings were z-transformed (considering test and filler items) to eliminate any inter-individual scaling differences. On the z-transformed scale, each participant has a mean of zero and a SD of one. The untransformed ratings had a grand mean of 3.56 and a standard deviation of 2.25, so that raw means per condition can be derived using 3.56+2.25 × *z-score*.

### Results

Table 1 shows the z-scored rating data for the test items, broken down by condition. Higher numbers represent a higher degree (or certainty) of perceived anomaly. As can be seen, the different types of violations (semantic, syntactic, rhyme, and metrical) were all reliably detected by participants, as reflected in considerably higher anomaly ratings for these conditions compared to the control condition. However, the rhyme violation condition received by far the highest anomaly ratings, supporting the assumption that rhyme violations are the most salient type of violation in the context of a Limerick. Paired *Bonferroni* t-tests (by participants and items) confirmed that each violation condition differed reliably from the control condition and that the rhyme violation condition was rated as more anomalous than any other condition (all *p*s <.01).

**Table 1:** Mean z-transformed anomaly ratings per condition ±95% CIs by subjects and items.

	Mean	95% CI by subjects	95% CI by items
Control	−0.74	±0.15	±0.20
Semantic	+0.25	±0.24	±0.25
Syntactic	+0.46	±0.21	±0.23
Rhyme	+1.06	±0.20	±0.14
Metric	+0.15	±0.19	±0.25

Table 2 shows the mean response times per condition (measured from the appearance of the 7-point scale on screen until one of the number keys was pressed). Paired *Bonferroni* t-tests (by participants and items) indicated that responses were made significantly faster in the control condition than in any of the violation conditions (*p*s <.05). Response times for the latter did not reliably differ from one another (*p*s >.5), suggesting that the speed of anomaly detection was independent of the kind of anomaly being detected.

**Table 2:** Mean response times (in ms) per condition ±95% CIs by subjects and items.

	Mean	95% CI by subjects	95% CI by items
Control	1740	±190	±242
Semantic	2279	±194	±245
Syntactic	2409	±192	±242
Rhyme	2273	±190	±239
Metric	2346	±191	±241

### Discussion

The purpose of Experiment 2 was to distinguish between two possible explanations for the pupil size data in Experiment 1: Rhyme violations might either evoke a qualitatively unique response in comparison to the other types of expectancy violations, or they just constitute the only clearly detectable type of violation in the context of a Limerick. Experiment 2 revealed that all types of violations were reliably detected compared to the control (no violation) condition; this supports the idea that pupil responses in the rhyme violation condition might reflect a qualitatively unique response. On the other hand, rhyme violations received by far the highest anomaly ratings, suggesting that it just requires a very salient kind of violation for a pupillary response to emerge.

**Figure 3:**
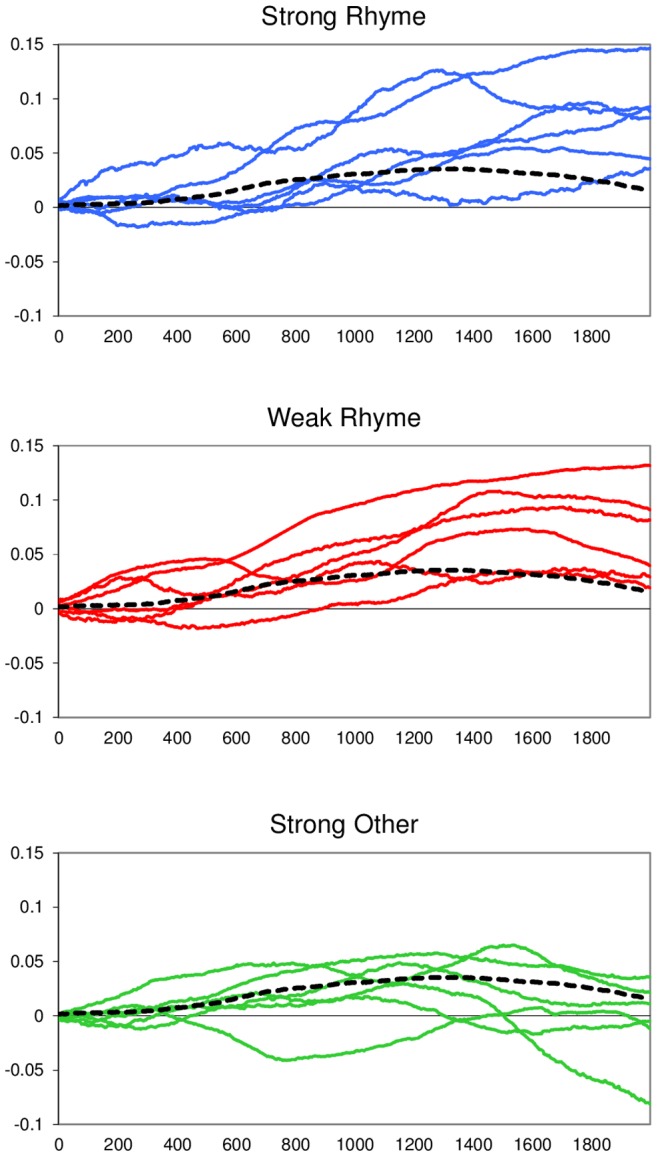
Pupil size data for extreme subsets of items. Pupil size (Y-axis) is measured in log number of pixels per video frame, relative to 200 ms pre-onset baseline. Time (X-axis) is sampled at 500 Hz from 0–2000 ms after critical word-onset. “Strong rhyme” (blue curves, top) shows data for the six items with the *highest* anomaly ratings in the rhyme violation condition; “weak rhyme” (red curves, middle) shows data for the six items with the *lowest* anomaly ratings in the rhyme violation condition; “strong other” (green curves, bottom) shows data for the six items with the *highest* anomaly ratings in any of the non-rhyme violation conditions. For reference, the grand average curve for the no violation control condition is also included in each plot (black dotted lines).

To further adjudicate between these two possibilities, we re-analyzed the pupil size data from Experiment 1, this time focusing on extreme subgroups of items based on the anomaly ratings from Experiment 2. Figure 3 shows pupil size plots for the six items with the *highest* anomaly ratings in the rhyme violation condition (“strong rhyme”; mean anomaly z-score = 1.46; range across items: 1.40–1.50; range across individual trials: 1.08–2.32), the six items with the *lowest* anomaly ratings in the rhyme violation condition (“weak rhyme”; mean anomaly z-score = 0.59; range across items: 0.30–0.75; range across individual trials: −0.92–1.53), and finally, the six items with the highest anomaly ratings in any of the non-rhyme violation conditions (“strong other”; mean anomaly z-score = 1.29; range across items: 1.13–1.65; range across individual trials: 0.66–3.25); the grand average for the control condition (no violation) is also included in Figure 3 as a reference. Two-tailed Mann-Whitney U Tests (by items and by individual trials) confirmed that the “weak rhyme” group of items had significantly lower anomaly scores than both the “strong rhyme” and “strong other” group of items (*p*s <.005); the latter two did not reliably differ in terms of perceived anomaly (*p*s >.1). Note that the Mann-Whitney U test is not only appropriate for small sample sizes but also robust against outliers.

Figure 3 suggests that salience of an anomaly alone may not be a good predictor of changes in pupil size. Rhyme violations evoked a clear, positive pupillary response *regardless* of whether they obtained a very high or a relatively moderate anomaly score in Experiment 2. By contrast, “strong other” violations (non-rhyme violations that obtained very high anomaly scores in Experiment 2) did not seem to evoke this kind of response – if anything, pupils appeared slightly less dilated for these stimuli relative to the control condition. Figure 3 shows that this pattern is fairly consistent across items in each subgroup. Indeed, after averaging pupil sizes over a time period of 1000–2000 ms from critical word-onset (as in the earlier analyses), there was a significant difference between the “strong rhyme” and “strong other” group of items (*p*<.03 by two-tailed Mann-Whitney U Test), a significant difference between the “weak rhyme” and “strong other” group of items (*p*<.04), and no difference between the “strong rhyme” and “weak rhyme” group of items (*p*>.5). In conclusion, our data do not support the assumption that pupil responses are merely a reflection of the strength or salience of an expectancy violation.

## General Discussion

The first experiment reported in this paper is the first to examine changes in pupil size as a function of *semantic*, *syntactic*, *rhyme*, and *metric* expectancy violations during spoken Limerick appreciation. It was found that only rhyme violations – but not semantic, syntactic, or metrical violations – elicited a significant pupillary response relative to the control condition (no violation). The response started about 200 ms after the non-rhyming word had been processed. The second experiment, based on anomaly ratings, established that all types of expectancy violations were reliably detectable relative to the control condition, and that rhyme violations were, on average, perceived as most severe. Interestingly, a reanalysis of pupil size data for extreme subgroups of items (determined via the anomaly ratings from Experiment 2) suggested that pupillary responses in the rhyme violation condition were unlikely to be due to the salience of the perceived anomaly alone. Rather, rhyme violations in Limericks appeared unique in the sense that they not only resulted in exceedingly high average anomaly ratings, but also in a reliable pupillary response compared to conditions involving other types of expectancy violations.

This raises the question of what the pupillary response in the rhyme violation condition actually reflects. Given the nature of the expectancy violations examined here, it seems unlikely that it reflects an increase in processing load comparable to that established in previous psycholinguistic research (e.g., [13], [14]). In those studies, pupillary responses were found to be sensitive to so-called *syntactic garden path* effects, triggered by revisions of local syntactic misanalyses (e.g., upon hearing “*was sleeping*” in “*while the mother dressed the baby was sleeping in the crib”*, cf. [14]). By contrast, the expectancy violations examined in the present experiments (including the rhyme violations) were not repairable in this sense – they were outright violations akin to those in, e.g., [9]. This may render a processing load interpretation of our pupil size data less feasible.

A perhaps more plausible candidate for explaining our results could be an *emotional* response to rhyme violations in Limericks, above and beyond anomaly detection *per se* (the latter mainly relies on metalinguistic judgement without necessitating emotional involvement). Pupillary responses have previously been shown to be sensitive to affective arousal independent of positive or negative emotional valence [15]. It is in the very nature of poetry appreciation that linguistic processing and emotional involvement are closely intertwined. Although poetry can and does make use of simple language (and Limericks normally do so, although with occasional rare diction), it characteristically produces emotional responses by relatively complex uses of language, such as double meanings or extra-syntactic semantic connections (for example, between rhyme words). Furthermore, attentive reception of poetry often not only involves *having* emotional responses, but also *reflection upon* those responses and their relationship to the poem. The emotional response to a Limerick with a missing rhyme at the end may be characterized as increased arousal due to a conflict arising between the perceiver’s expectation of a rhyme and the actual spoken input. The affective evaluation of this conflict may be more negative in some instances (‘a poorly constructed Limerick’) and more positive in others (‘an original departure from the norm’).

In addition to the issue of emotionality, there remain at least two further questions for follow-up research. First, why do the present results differ from the null result with regard to the effects of rhyme violation reported in [3]? One possibility is that the previous study had insufficient statistical power, especially in the light of the rather similar conditions that were compared. Related to this, pupil diameter may be a more sensitive measure compared to the reading efficiency scores in [3], especially with regard to evaluative aspects of poetry processing. Another explanation could be the fact that we changed from a visual to an auditory mode of poetry presentation. As we have mentioned in the Introduction, several features of poetry were originally devised to support auditory communication, and the change towards an auditory presentation mode (including its potentially stronger emphasis on phonological processing) may well have enhanced the effects of rhyme violations in the present study. Last but not least, Limericks and the poems investigated in [3] belong to different sub-genres of poetry whose cognitive processing and emotional evaluation may be differentially affected by rhyme violations.

The second open question is why there were no clear effects of semantic violations on pupil size despite the strong evidence for semantic violation effects on nervous activity (see Introduction). One possible answer comes from Jakobson (1966, cited in [10], p. 342) who stated that poetry does not have to strive for a maximum degree of semantic coherence when compared to ordinary prose. Instead, a firm prosody (i.e. the rhyme scheme and metre) can serve to connect the elements of a poem. This view appears to be supported by the present results. Note that Kutas and Hillyard [9] did not present their stimuli in a poetry context; Hoorn [10] did present poems and found the strongest effect in ERP when semantic violations were combined with rhyme violations. In the present study, we tried to ‘orthogonalize’ the different types of violations as much as possible, but our prediction for future research would be that combined semantic and rhyme violations should have at least additive effects on processing.

In conclusion, pupil size changes during spoken Limerick appreciation suggested a close link between the detection of rhyme violations on the one hand and their emotional evaluation on the other, opening up interesting avenues for further research in the area of poetry processing.

## Supporting Information

Appendix S1 **Experimental materials.**
(DOC)

Appendix S2 **Illustration of the smoothing and interpolation procedures used in Experiment 1.**
(DOCX)
