# Supplementary material for: Listening to Limericks: A Pupillometry Investigation of Perceivers’ Expectancy
Source: PLoS One. 2013 Sep 23;8(9):e74986. doi: 10.1371/journal.pone.0074986 (PMC3781151; doi:10.1371/journal.pone.0074986)
Supplement: Appendix S1 — Experimental materials. (DOC) [file pone.0074986.s001.doc]

# Material Appendix

*Listening to Limericks: A pupillometry investigation of perceivers’ expectancy*

Scheepers, Mohr, Fischer, & Roberts

Listed below are transcripts of the 25 spoken Limericks used as experimental stimuli. The corresponding sound recordings can be downloaded at:

<http://www.psy.gla.ac.uk/~christop/LimerickAppendix/>

Item numbers of the transcripts correspond to those in the sound files. The last five lines per transcript are ordered according to the five different versions per item: *control* (no violation), *semantic violation*, *syntactic violation*, *rhyme violation*, and *metric violation*. In the sound files, the different versions are labelled *a*, *b*, *c*, *d*, and *e*, respectively.

1

As Londoners say, in Calcutta

Lives a man with a terrible stutter.

When he asks for the bread,

They will pass him instead:

Beer, broccoli, beans and the butter.

Beer, broccoli, beans and the gutter.

Beer, broccoli, beans some and butter.

Beer, broccoli, beans and the biscuits.

Beer, broccoli, beans and also butter.

2

There was a nice carrot, quite meek

Went boating one day for a week

But alas and alack!

He never came back

‘Cos he got on a boat with a leek.

‘Cos he got on a boat with a week.

‘Cos he got on a boat a with leek.

‘Cos he got on a boat with a bean.

‘Cos he got on a boat with a huge leek.

3

There once was an old man of Esser,
Whose knowledge grew lesser and lesser,
It at last grew so small
He knew nothing at all,
And now he's a college professor.

And now he's a college compressor.

And now he's for college professor.

And now he's a college assistant.

And now he's a university professor.

4

A mouse in her room woke Miss Doud
Who was frightened and screamed very loud
Then a happy thought hit her
To scare off the critter
She sat up in bed and just meowed.

She sat up in bed and just ploughed.

She sat up in bed to just meowed.

She sat up in bed and just purred.

She sat up in bed and loudly meowed.

5

There was an old man of the coast

Who placidly sat on a post

But when it was cold

He relinquished his hold

And called for some hot, buttered toast.

And called for some hot, buttered ghost.

And called for hot, buttered some toast.

And called for some hot, buttered bread.

And called for some buttered toast.

6

A lady from South Carolina

Took a cruise on a great ocean liner.

She said to the stoker,

Who was a bit of a joker,

“It’s high time you saw Indo-China”.

“It’s high time you saw my angina”.

“It’s high time you seen Indo-China”.

“It’s high time you saw Madagascar”.

“It’s high time you saw China”.

7

There was an old soldier of Bistor

Was walking one day with his sister;

A bull, with one poke,

Tossed her into an oak,

Before the old gentleman missed her.

Before the old gentleman missed fur.

Before the old gentleman miss her.

Before the old gentleman missed Jane.

Before the old gentleman ultimately missed her.

8

Just had a plate of veg broth,

Spilt some and needed a cloth.

My Ribena was good,

I drank what I could,

Then blew bubbles and made lots of froth.

Then blew bubbles and made tiger moth.

Then blew bubbles and made of lots froth.

Then blew bubbles and made lots of foam.

Then blew bubbles and made lashings of froth.

9

There was an old woman in Surrey,

Who was morn, noon, and night in a hurry,

Called her husband a fool,

Drove her children to school,

Before dashing to work in a flurry.

Before dashing to work in a curry.

Before dashing to work a in flurry.

Before dashing to work in a rush.

Before dashing to work in a horrendous flurry.

10

There was an old miser at Reading,

Had a house, and a yard with a shed in;

‘Twas meant for a cow,

But so small, that I vow

The poor creature could scarce get its head in.

The poor creature could scarce get its bed in.

The poor creature could scarce its get head in.

The poor creature could scarce get its head through.

The poor creature could scarcely get its head in.

11

As a little fat man of Bombay,

Was smoking one very hot day,

A bird called a snipe,

Flew away with his pipe,

While his wife cheered hip hip and hooray!

While his wife cheered hip hip and Monday!

While his wife cheered hip and hip hooray!

While his wife cheered hip hip and hurrah!

While his wife cheered hip hip hooray!

12

A snowman who lived in Tibet,

Bought some snow shoes, to keep out the wet.

“They’re the most”, he declared,

“Peculiar and weird,

And abominable shoes I could get.”

And abominable shoes I could sweat.”

And abominable shoes could I get.”

And abominable shoes I could find.”

And abominable shoes I could finally get.”

13

There was a young lady from Wemyss,

Who, it seems, was troubled with dreams.

She would wake in the night

And, in terrible fright,

Shake the beams of the house with her screams.

Shake the beams of the house with her gleams.

Shake the beams of the house her with screams.

Shake the beams of the house with her shouts.

Shake the beams of the house with anguished screams.

14

There once was a dashing young mouse

Who was bored with only one spouse.

“I think one more wife,

Would add spice to my life,

And be nicer to have round the house.”

And be nicer to have round the blouse.”

And be nicer to have the round house.”

And be nicer to have round the flat.”

And be nicer to have around the house.”

15

A gentleman, dining at Crewe,

Found quite a large mouse in his stew.

Said the waiter, “Don’t shout,

And wave it about,

Or the rest will be wanting one, too!”

Or the rest will be wanting canoe!”

Or the rest will be one wanting, too!”

Or the rest will be wanting as well!”

Or the rest will be wanting their own, too!”

16

There was a young lady of York,

Who ate pears through her ears with a fork;

She picked peas with her toes

To her knees, then her nose,

‘Cos they’d stoppered her mouth with a cork!

‘Cos they’d stoppered her mouth with a pork!

‘Cos they’d stoppered her mouth a with cork!

‘Cos they’d stoppered her mouth with a plug!

‘Cos they’d stoppered her mouth with a big cork!

17

A bachelor living in Fife

Decided he needed a wife.

He met a nice girl

And proposed in a whirl,

But she answered: “Huh! Not on your life!”

But she answered: “Huh! Not on your knife!”

But she answered: “Huh! On your not life!”

But she answered: “Huh! Not on your luck!”

But she answered: “Huh! Not on your own life!”

18

There was a young lady called Lynne

Who was so excessively thin

That when she essayed

To drink lemonade

She slipped through the straw and got in.

She slipped through the straw and got gin.

She slipped through the straw got and in.

She slipped through the straw and got wet.

She slipped through the straw and then got in.

19

A bugler named Edward MacDougal

Found ingenious ways to be frugal.

He learned how to sneeze

In various keys,

Thus saving the price of a bugle.

Thus saving the price of a google.

Thus saving the price a of bugle.

Thus saving the price of a horn.

Thus saving the price of a new bugle.

20

I know there's an issue with odds,

But the answer is known to the Gods.

So the people who win

Either abstain from Sin

Or are nothing but rich lucky sods.

Or are nothing but rich lucky squads.

Or are nothing rich lucky but sods.

Or are nothing but rich lucky men.

Or are nothing but rich and lucky sods.

21

There once was a young man called Scott,

Whose poems made me laugh a lot,

Much better than mine,

Which quite clearly don't rhyme,

It's a talent that I haven't got.

It's a talent that I haven’t shot.

It's a talent that haven't I got.

It's a talent that I just don’t have.

It's a talent that I just haven't got.

22

On Monday at just after five

She felt she was buried alive

I'm quite smart enough

To finish this off

But somehow I don't have the drive.

But somehow I don't have the five.

But somehow I don't the have drive.

But somehow I don't have the push.

But somehow I don't really have the drive.

23

There once was a young man called Phil

Who said: “I am feeling quite ill…

Took my girl for a meal

To say how I feel.

But now, I am paying the bill!”

But now, I am paying the drill!”

But now, I am the paying bill!”

But now, I am paying the cheque!”

But now, I am paying the whole bill!”

24

A young man, while learning to ski,

Cried out, “Alas! Woe is me!

I’ve done all the jumps

Over various humps

And I always end up in a tree!”

And I always end up in a tea!”

And I always end in up a tree!”

And I always end up in a spruce!”

And I always end up in a huge tree!”

25

There once was a slimy big slug

Who chose to live under a rug.

He loved to drink beer

And that’s how, I fear,

He ended his life in a mug.

He ended his life in a bug.

He ended his life in with mug.

He ended his life in a glass.

He ended his life in an empty mug.
