# Supplementary material for: Listening to Limericks: A Pupillometry Investigation of Perceivers’ Expectancy
Source: PLoS One. 2013 Sep 23;8(9):e74986. doi: 10.1371/journal.pone.0074986 (PMC3781151; doi:10.1371/journal.pone.0074986)
Supplement: Appendix S2 — Illustration of the smoothing and interpolation procedures used in Experiment 1. (DOCX) [file pone.0074986.s002.docx]

# Smoothing/Interpolation

*Listening to Limericks: A pupillometry investigation of perceivers’ expectancy*

Scheepers, Mohr, Fischer, & Roberts

Illustration of the smoothing and interpolation procedure applied to the 37.2% of trials with missing pupil size data within the critical 2200 ms time period. The panels on the left show original data for three randomly chosen trials with missing pupil size samples. The missing data resulted from blinks or—very occasionally—small corrective saccades, which were removed prior to analysis. The panels on the right show the same data after applying the non-linear interpolation method. The procedure (*2^nd^ order B-spline estimation* after *Loess* smoothing over 3% data windows) was carried out in TableCurve 2D v5.01 (SYSTAT Software Inc., 2002).
